# Actuation of Cell Layers in Three Dimensions

**DOI:** 10.1002/adma.202522191

**Published:** 2026-05-13

**Authors:** Kirsten Endresen, Aniruddh Murali, Birte C. Geerds, Grecia M. Valenzuela Portillo, Maria Bloksgaard, Daniel J.G. Pearce, Francesca Serra

**Affiliations:** ^1^ Dept. Physics and Astronomy Johns Hopkins University Baltimore Maryland USA; ^2^ Dept. Physics, Chemistry and Pharmacy University of Southern Denmark Odense Denmark; ^3^ Dept. Theoretical Physics University of Geneva Geneva Switzerland

**Keywords:** 3D structure, liquid crystal elastomers, nematic order in cells, tissue patterning

## Abstract

The alignment of fibers and cells in living tissues affect their mechanical properties and functionality. In this context, one can draw an analogy between tissues and nematic liquid crystal elastomers. We explore this analogy by growing fibroblasts on 2D‐patterned substrates and observing the contraction of cell sheets upon detachment from the substrates. When fibroblast sheets detach, they undergo an anisotropic contraction, with maximum contraction along the nematic director, like nematic elastomers do during phase transition. We quantify this anisotropy using substrates patterned with stripes to induce alignment, finding that cell sheets resemble nematic elastomers with negative 2D Poisson ratio. The contraction of the peeling sheet is robust to drugs that modulate cytoskeletal remodeling. We then apply design principles used for programming curvature in nematic elastomers to actuate 3D structures in the detached fibroblast layers, demonstrating an application of these principles and we support the results with simulations. This proof of concept shows the ability to control the 3D shape through 2D patterning in cell layers, leading to promising avenues to program tissues.

## Graphical abstract

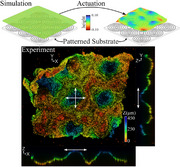

## 1 Introduction

Morphogenesis, the generation of shape in biological tissues, is a complex phenomenon that happens due to the interplay of chemical, biological and physical cues [1, 2]. Evidence is gathering that among the physical cues, cells' nematic alignment plays an important role [3, 4, 5, 6, 7]. In addition, the generation of tissues with desired target shapes through the self‐assembly and self‐organization of cells is an appealing idea for the creation of implants with maximal adaptability to the target organ. Existing strategies to obtain tuneable implants rely on the creation of stimuli‐responsive environments such as gels or bioprinted scaffolds [8, 9, 10, 11]. Examples include soft materials sensitive to osmotic pressure [12], chemicals [13], mechanical stimuli [14], magnetic fields [15], temperature [16], light [17, 18], or pH [19]. In all these applications the scaffold itself, either a porous hydrogel or a structure fabricated with a precise geometry (e.g. via 3D printing), modifies its shape or its mechanical properties such as stiffness in response to an external stimulus. Another less common approach to tissue engineering is the layer‐by‐layer approach [20]. This consists of harvesting layers of cells grown on stimuli‐responsive surfaces, for example using thermoresponsive polymers, or using enzymes that cleave the cell‐substrate links. The layer‐by‐layer approach allows for a more precise control of cell alignment [21]. The alignment of cells has been highlighted as an important factor for structure and functionality [22, 23]. For example, host acceptance of cardiac implants or other bioprinted tissues improves if the orientation of the cells matches the orientation of the surrounding tissue [24, 25, 26]. In biotissues built by layer‐by‐layer methods, the alignment of cells can be controlled in 2D cultures and then used in 3D multi‐layered structures [20, 27, 28].

The analogy between spontaneously aligning cells and nematic liquid crystals can provide relevant insight [29]. Cell alignment influences cell–cell communication, cell migration, and even morphological features and differentiation. For example, by analyzing topological defects in the nematic order of cells, it was shown that defects impact the rate of cell death [30], the formation of protrusions from a flat cell layer [31, 32, 33, 34, 35, 36] and the potential for tissue regeneration [37].

An interesting aspect of this analogy comes into focus when one considers the effect of the liquid crystal order on the mechanical properties of tissues, in particular by considering tissues as a special type of liquid crystal elastomer. Liquid crystal elastomers are polymer networks with liquid crystal mesogens embedded in the polymer main or side chains [38]. One of the consequences of their mesogenic nature is the ability to change their macroscopic shape in response to a phase transition, due to the coupling between nematic order and mechanical properties.

The ability to respond to stimuli, often called actuation, is a consequence of such coupling. This is exemplified by the simple observation that the equilibrium shape of a uniformly aligned nematic elastomer changes in the transition from the nematic to the isotropic phase. If the elastomer is crosslinked in the aligned nematic phase, at the phase transition it contracts along the nematic director and expands in the directions perpendicular to it. By imposing an initially non‐uniform alignment, for example including topological defects, this anisotropic expansion/contraction can result in generation of Gaussian curvature [39, 40, 41, 42, 43].

This property is intriguing in the context of cell sheets. In particular, populations of fibroblasts grown in 2D at high density display nematic order [44, 45]. Moreover, as cells proliferate they secrete polymers that create an extracellular matrix (ECM), an elastic medium in which the cells are embedded [46]. The nematic order of the cells favors a certain degree of alignment of the elastic matrix [47]. We could therefore expect that this composite elastic medium behaves in a similar manner to liquid crystal elastomers, where the presence of topological defects is associated with generation of Gaussian curvature. To test this hypothesis, we analyze the behavior of layers of fibroblast cells, whose alignment is controlled by a topographic pattern on the substrate.

## 2 Results

### 2.1 Uniaxial Contraction

**FIGURE 1:**
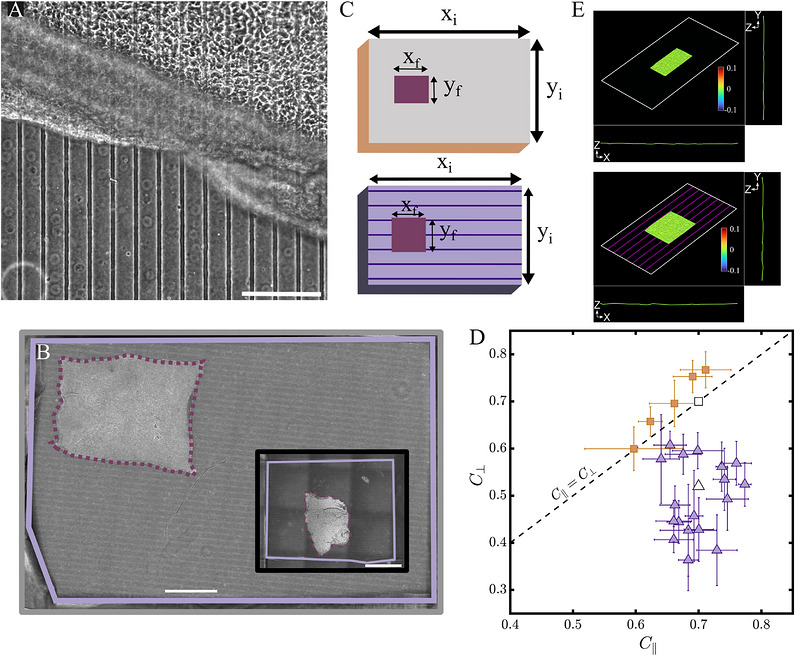
Detachment of cell sheets from striped and unpatterned PDMS. (A) Phase contrast microscopy image of a cell sheet peeling off a PDMS slab with striped pattern (*h* = 2 μm, *w* = 60 μm). The bottom part of the image shows the substrate after cells have peeled off, while in the top part cells are still attached. Scale bar 200 μm. (B) Image of an entire PDMS slab (outlined in lavender) after detachment is complete. The layer of cells, after peeling, is a smaller flat rectangle outlined in maroon dots. Scale bar 1000 μm. Inset: sheet peeled from a small slab of PDMS with striped pattern after exposing the sample to Y27632 (30 min, 10 μM) prior to peeling. (C) Schematic of the cell sheet peeled from PDMS with plain stripes (top) and parallel (bottom). For the striped pattern $x_{i}$ and $y_{i}$ are the initial dimensions of the PDMS sample parallel and perpendicular to the stripes, respectively. $x_{f}$ and $y_{f}$ are the final dimensions of the peeled sheet along the corresponding axes. For the plain PDMS $x_{i}$ and $y_{i}$ are defined as the dimensions of the longer and shorter axes of the PDMS, respectively, and $x_{f}$ and $y_{f}$ are the final dimensions of the peeled cell sheet along the corresponding axes. (D) Contraction of the cell sheet parallel and perpendicular to the alignment of the cells. Sample data from plain PDMS (here, $C_{∥}$ indicates the contraction along the long axis of the sample) are indicated in orange squares and and data for striped PDMS are indicated in purple triangles. The black dotted line corresponds to isotropic contraction ($C_{∥}=C_{⊥}$). Errors and statistical analysis are described in Methods and Supporting Information. The white square and triangle indicate the contraction for isotropic or aligned samples, respectively, obtained via simulation. (E) Final shape of simulated cell sheets without stripes (top) and with (bottom) and their height profiles measured along a cross‐section through the center in x‐ and y‐direction respectively. The color indicates the vertical deviation of the sheets with both remaining largely flat. Purple stripes in the bottom panel represent the patterning. See also Movie S1.

We grow NIH‐3T3 mouse fibroblasts to a high density on slabs of polydimethylsiloxane (PDMS), treated with poly‐D‐lysine, a common polypeptide for cell culture. At very high density, after a few days of growth, a flexible skin made of fibroblasts and ECM tends to spontaneously detach from the substrate [48, 49], peeling off starting from the edges of the sample. Any perturbations to the environment, such as shaking or changing growth media, triggers such detachment. A similar effect can also be induced by degradation of the substrate gel [13] or with the use of thermoresponsive polymers [50]. In our experiments, we drag a scalpel blade along the perimeter of the slab. As soon as the edges of the cell layer are released from the PDMS, the detachment of the whole layer proceeds spontaneously throughout the sample (Figure 1A and Figure S1). Upon detachment, we notice that most fibroblasts are in the floating sheet and only very few fibroblasts are left on the PDMS substrate. The area of the detached cell sheet is significantly smaller than the initial area occupied by the cells on the substrate (Figure 1B), showing auxetic behavior with contraction along both dimensions in 2D.

Figure 1A shows a phase contrast image of cells (microscope Nikon Ti‐2 Eclipse) during peeling from PDMS substrates patterned with parallel ridges, or stripes, 10 μm wide and 2 μm tall, spaced by 60 μm. One side of the PDMS is fixed at approximately 7.5 mm, with the opposing side ranging from 2.5 to 5 mm. We observe that the sheet often starts to detach from an edge or a corner and lift over the course of several minutes (Figure S1). This time scale, in the order of minutes, is consistent with cytoskeletal remodeling dynamics underlying cell attachment and detachment [51] and with tissue stretching experiments [52, 53]. The total time for the peeling of a sample varies between samples from 5 to 25 min and this variability is due to the presence of points where cells are pinned to the surface and the peeling gets stalled. An example of this is shown in Figure S2.

Peeling continues after gently pushing the cells past the pinned points. Figure 1B shows the final stable shape of a detached cell sheet (outlined in maroon dotted line). The cell sheet retains the flat, rectangular shape of the substrate (outlined in lavender) albeit with jagged edges. The stable shape suggest it has undergone a plastic deformation whereas the reduced size and changed aspect ratio imply anisotropic contraction.

On the ridges NIH‐3T3 cells reach confluency with uniform nematic alignment featuring a director oriented along the stripes (also shown in Figure S1.A). We can see from microscopy that although there are some height fluctuations across the sample, the cell sheets remain essentially flat. Sometimes the peeled samples' corners may fold inward creating a deformation in the margin, due to the movement of the liquid above the floppy cell sheet. Similar rolling and folding of cell sheets have been reported by Kawecki et al. [54], highlighting the fragility of fibroblast sheets. In order to minimize the effect of shearing from the fluid above the sheet, we reduce the volume of cell media prior to peeling and take precautions to move the sample very slowly and carefully. Even with all these precautions, about 10% of the samples roll up or fold. The sheets that fully roll into tubes, or that rupture due to the fluid movement, are discarded in our analysis.

For comparison, we perform the peeling experiments also on the cells exposed to different types of drugs targeting the actin cytoskeleton or other properties of the cells (details in Methods), as shown in Figure S3 and in the inset of Figure 1B. With the exception of trypsin, which cleaves peptide bonds and thereby cell–cell and cell‐substrate connections, and paraformaldehyde, which fixes the cells completely, drugs that interfere with actin remodeling (Y27632 shown in Figure 1B inset [55], mycalolide B [56], cytochalasin D [57], and a “freeze actin” mixture [58] composed of latrunculin A, jasplakinolide and Y27632) do not prevent the peeling from happening or the cell layer from shrinking. Further, we verify that, as expected, at this stage of cell development the presence of collagen I in ECM is below detection limit and collagen IV is sparse [59] (Figure S4).

If the sheet detaches without rolling, as in Figure 1B or Figure S5 (for plain substrate), we can measure the contraction from 2D phase contrast images, combined together with the ImageJ stitching plugin [60]. We take as reference the dimensions of the PDMS to determine the initial dimensions of the cell sheet ($x_{i}$ and $y_{i}$) and measure the final dimensions of the sheet ($x_{f}$ and $y_{f}$), as shown in the schematics of Figure 1C. Without loss of generality, we choose the $\hat{x}$ direction to be aligned with the stripes.

We estimate the fractional contraction along the nematic director ($C_{∥}$) and the fractional contraction perpendicular to the director ($C_{⊥}$) as $C_{∥}=\frac{x_{i}-x_{f}}{x_{i}}$ and $C_{⊥}=\frac{y_{i}-y_{f}}{y_{i}}$, respectively. The results are shown in Figure 1D (purple triangles), where each point represents the measured total contraction for one cell sheet and the error bars indicate uncertainties in the measurements that arise from their irregular shapes (as detailed in Methods). The data consistently show that for the cell sheets that detach from striped PDMS the contraction along the stripes direction is greater than the contraction perpendicular to the stripes. The average $C_{∥}$ observed among these samples is 0.70±0.04, whereas the average $C_{⊥}$ is 0.49±0.07. The difference is apparent from the visible change in the aspect ratio of the samples.

We can compare this with cells grown on plain rectangular blocks of PDMS, which do not impose any anisotropy onto the cells. Here we define $C_{∥}$ as the contraction along the longest edge of the sample. In the absence of stripes, we observe isotropic contraction ($C_{∥}\approx C_{⊥})$, see Figure 1D (orange squares). The average $C_{∥}$ among these samples is 0.66±0.05, whereas the average $C_{⊥}$ is 0.69±0.07. If we compare these results to the ones obtained from striped patterns, we notice that (i) the contraction parallel to the stripes is similar to the contraction of the cells on plain PDMS, but (ii) the contraction perpendicular to the stripes is significantly reduced. Moreover, (iii) there is a large difference in the contraction along and perpendicular to the stripes (see Supporting Information). We analyze these effects in the Discussion section.

The anisotropic contraction suggests that the cell sheet behaves as an anisotropic elastic material. We then define the material's stretch factor as $\lambda =1-C_{∥}$, which gives the factor by which the material extends/contracts in the direction parallel to the cellular alignment. The stretch factor perpendicular to the alignment can be written as $1-C_{⊥}≔\lambda^{-\nu }$ where we have introduced the 2D Poisson ratio, ν. Using the measured contraction values ($C_{∥}$ and $C_{⊥}$) from flat sheets, we obtain λ=0.30±0.04 and ν=−0.58±0.14.

The data points in Figure 1D are taken by varying parameters such as the initial density of cells (the seeding density), the growth time and the initial area of the cell sheet, the aspect ratio of the PDMS slab on which the cells grow, as detailed in Tables S1 and S2. We analyze the data to compare the effects of cell growth time and slab aspect ratio on the measured contraction parameters (Tables S1 and S2). Within experimental uncertainty, cell growth time does not significantly affect either $C_{∥}$ or $C_{⊥}$, as confirmed by T‐tests (Table S3). By contrast, the initial slab aspect ratio seems to show some influences on the contraction along the perpendicular direction in striped samples, with higher aspect ratios leading to higher perpendicular contraction, consistent with previous observations [61]. A more systematic investigation of this effect will be required in future experiments. Finally, we can also test the viability of cells after detachment. As shown in Figure S6, if the detached cell sheet is deposited upon a petri dish, the cells will start to grow, showing that the cells remain alive during the process.

We can simulate the contraction of the cell sheets by considering them as thin elastic materials that, upon peeling, relax internal stresses causing them to deform, see Methods, Supporting Information and [62] for details. Crucially, this relaxation can be locally anisotropic, encoded by the orientation of the ridges on the PDMS substrate. We can measure the anisotropy by directly fitting our model to the experimental data in Figure 1D, see methods section for details.

Figure 1E shows the initial and final shape of a simulated cell sheet both with (top) and without (bottom) anisotropy (see also Movie S1). The white rectangular line indicates the initial size and the purple lines (where present) indicate the orientation of the ridges. The surfaces are colored by their vertical height with the XZ and YZ cross sections alongside, indicating no curvature is generated with or without stripes. Following the same protocol as in experiments, we measure $C_{∥}$ and $C_{⊥}$ in simulations, giving estimates of $C_{∥}=0.7$ and $C_{⊥}=0.52$ shown in Figure 1D. This corresponds to values of λ=0.3 and ν=−0.6 for our simulated cell sheets.

### 2.2 Nematic Order

Due to the many similarities, we opt to interpret our system using the framework of liquid crystal elastomers. In liquid crystal elastomers, ν is the opto‐thermal Poisson ratio and it relates the parallel and perpendicular changes in length during phase transitions. We first verify that for the cells the anisotropic contraction corresponds to a change in the degree of nematic order. We quantify the degree of order of the system before and after detachment by looking at the alignment of actin filaments. In previous work we and others have shown how the actin filaments follow the alignment of the fibroblasts [63, 64, 65]. Therefore, we utilize actin as proxy for cell nematic alignment and quantify the alignment of the actin network to have indication of the nematic order in our system.

**FIGURE 2:**
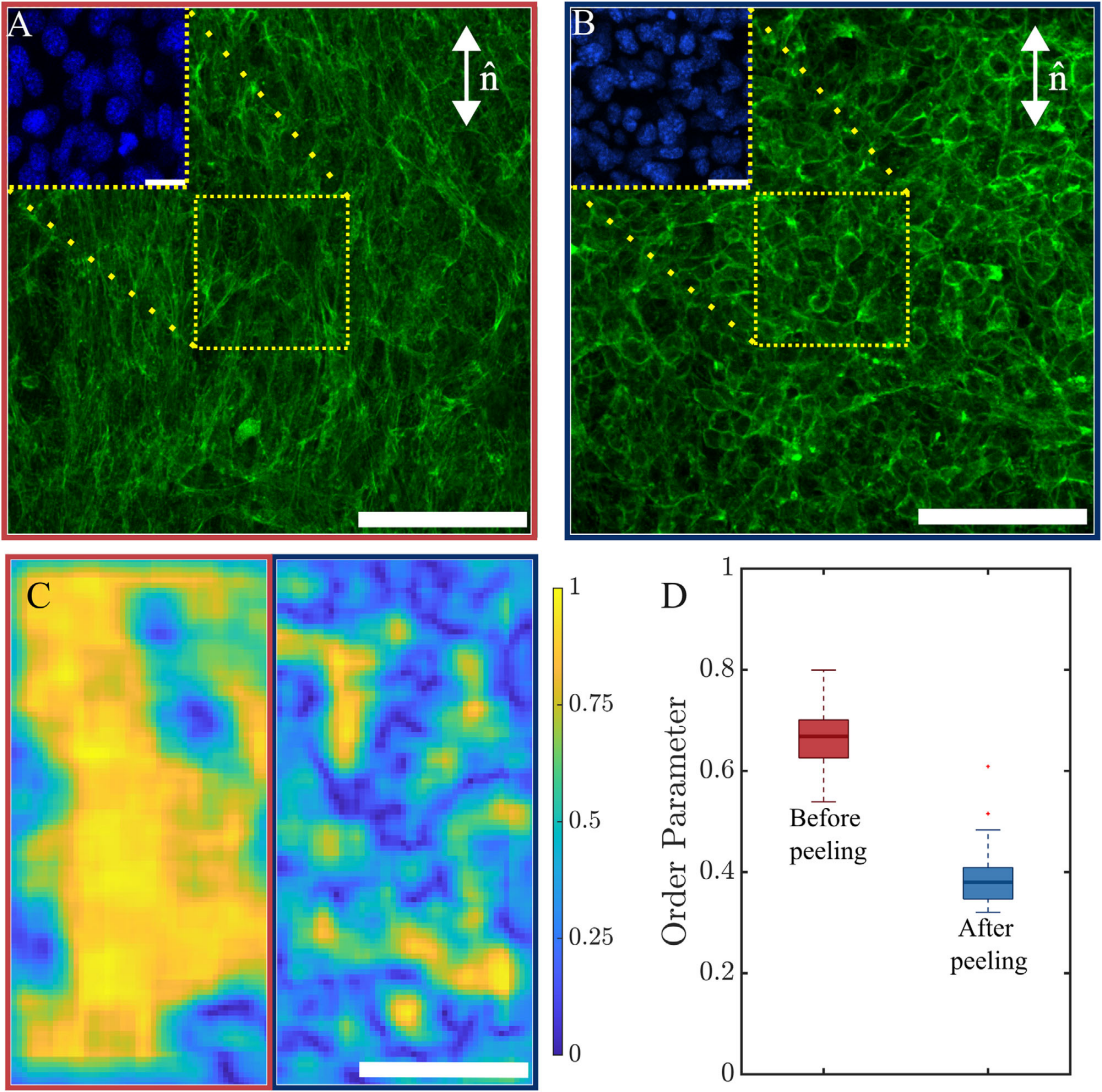
Order parameter change upon detachment. (A, B) Fluorescence image of actin filaments and nuclei (stained with Phallodin for actin and NucBlue Fixed Cell ReadyProbes for nuclei, in insets) before (A) and after (B) the peeling of cells. Scale bars 100 μm in figure and 20μm in insets. (C) Color maps of order parameter S measured from images (A) on the left and (B) on the right after the analysis with OrientationJ (only half the images are shown). (D) Box plot of order parameter as defined in the paper, and calculated over 2 different samples (total *n* = 21 images) before peeling and 2 different samples (total *n* = 31 images) after detachment.

We image the actin filaments stained with phalloidin rhodamine and nuclei stained with NucBlue fluorescent dye, and we analyze the fluorescence microscopy images using OrientationJ [66]. The results are shown in Figure 2, where we see the fluorescence images of the actin network before (Figure 2A) and after (Figure 2B) detaching, alongside the nuclear fluorescence (insets). The alignment direction of the ridges ($\hat{n}$) is shown in the figure, and it is evident that the actin appears more uniformly aligned in panel A, with the alignment direction matching that of the stripes. There is a significant loss of alignment when the cells are peeled from the substrate. These data can be qualitatively corroborated by observing the cell nuclei. When the cells are attached, nuclei are more sparse and more elongated, while when cells detach the nuclear density is higher and the nuclei are smaller and more disordered. We should remark that the high nuclear density makes it difficult to use nuclear fluorescence to accurately quantify the order parameter of cells.

We quantify the degree of alignment of the actin with a color map (Figure 2C) representing a local order parameter S defined as:

$$S=\sqrt{{\left\langle \cos2\theta \right\rangle }_{x,y\in W}^{2}+{\left\langle \sin2\theta \right\rangle }_{x,y\in W}^{2}}$$

where θ is the angle between vectors detected by OrientationJ within a square region W (following [44], see details in Methods). S is the largest eigenvalue of the 2‐dimensional nematic tensor (often called Q‐tensor), defined as $Q_{ij}=<u_{i}u_{j}-\frac{1}{2}\delta_{ij}>$, where u is a unit vector representing the orientation of mesogens and $\delta_{ij}$ is the Kronecker delta. In 2‐dimension, S ranges from 0 to 1, with 0 representing disorder and 1 representing perfect alignment. The local order, S, of cell layers on the striped substrates reduces after detachment as shown in Figure 2D. When the cells are supported by the textured substrate, it induces a degree of nematic alignment in the cells, indicated by the high S. When the cells are released from the substrate, the cells relax to a less aligned state, indicated by the drop in S. The drop in *S* is significant as shown in Supporting Information. Further analysis also shows that the order is not completely lost, as neither the local or global order parameter reach zero in the contracted state (see Figure S7).

By measuring the eigenvectors of the nematic tensor, we can also verify that the sample maintains a preferential alignment along the ridge direction even after the sample is detached. Even with these caveats, the data suggest that the peeling process is a way to induce a transition between a higher order and a lower order state, or to oversimplify between nematic and isotropic phase.

### 2.3 Actuation of Cell Layers

**FIGURE 3:**
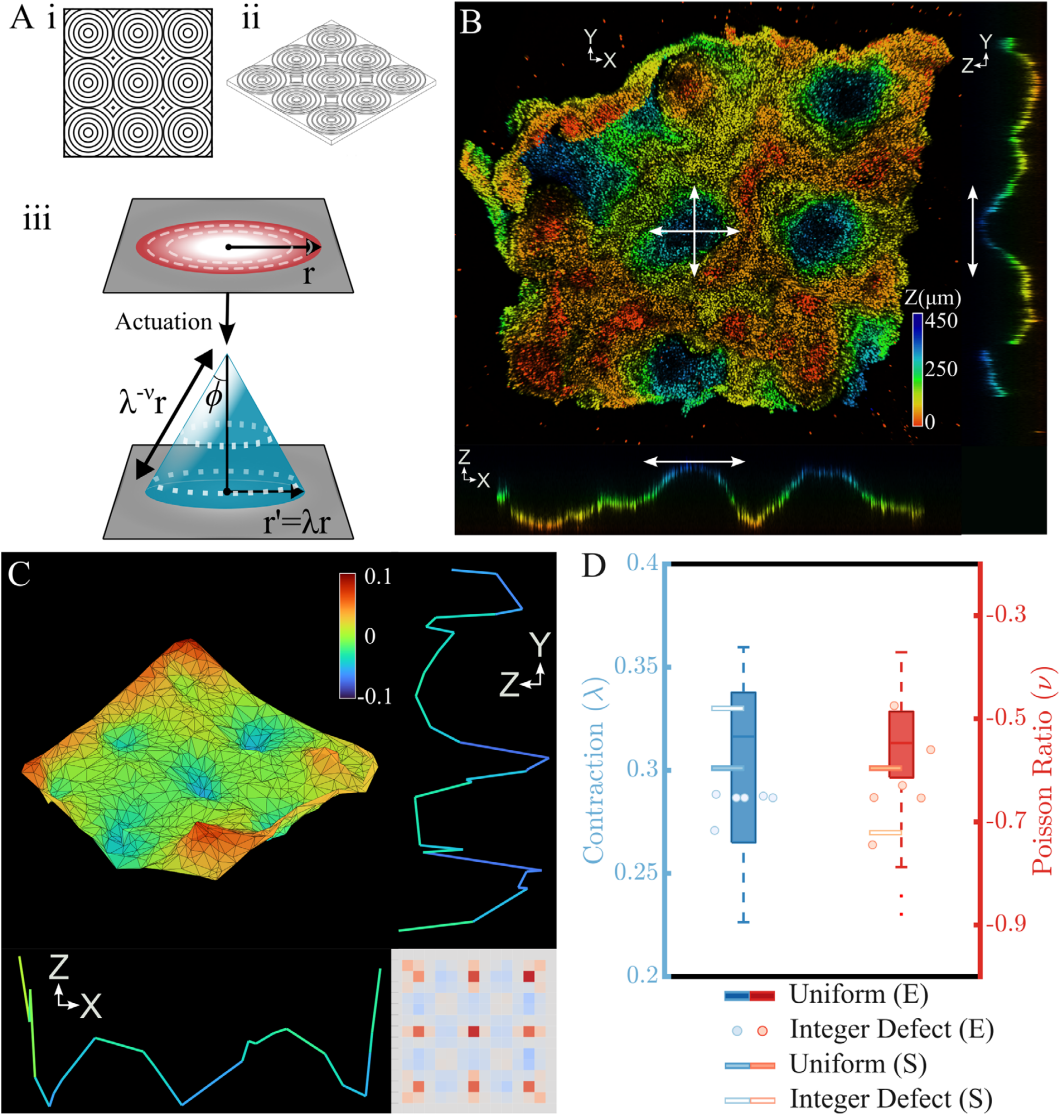
Actuation of cell sheets. Schematic of the defect array patterns used in experiments (i), and a 3D rendering of the substrate topography with the height of the ridges at 1.5–2 μm, ridge width 9 μm, and distance between the ridges is fixed at 60 μm (ii). (iii) Schematic of actuation in +1 topological defect where a circular region of initial radius r on a flat surface is actuated, causing an in‐plane contraction by a factor λ and an out‐of‐plane deformation into a conical shape with opening angle ϕ. The radial distance changes from r to $r^{\prime }=\lambda r$, while the slant length becomes $\lambda^{-\nu }r$. (B) Confocal microscopy image of a sheet of cells detached from the defect array. The overall dimensions of the peeled sample are 2000 μm× 2500 μm. The color bar shows the height difference. XZ and YZ cross‐sections along the directions indicated by white arrows reveal a sheet of uniform thickness with localized out‐of‐plane deformations. (C) Corresponding simulations of the cell sheet deformation. The inset on the bottom right corner represents a color map of Gaussian curvature (see Supporting Information for details and Movie S2). (D) Values of stretch factor λ and 2D Poisson ratio ν calculated from experiments (box plots for the flat samples with parallel ridges, and clear circles from curved samples with +1 topological defects) and simulations (filled rectangles for flat samples and clear rectangles for samples with +1 topological defects).

Since the cell sheets from striped substrates exhibit anisotropic contraction, as expected with a change of order parameter, we also test whether spatially varying alignment can generate Gaussian curvature. We first study a 2D array of +1/–1 topological defects, arranged in a square lattice, represented with an approximate schematic in Figure 3A, which we have extensively studied in previous work [64, 67, 68]. PDMS is cut into 3 × 3 grids of +1 defects (total size 7.5 mm × 7.5 mm), which introduces four −1 defects in between. It is known that in liquid crystal elastomers this pattern generates cones when actuated upon phase transition [69].

Figure 3B is a confocal microscopy image showing the structure of a cell sheet detached from a +1/–1 defect array, in which the cell nuclei were tagged with NucBlue Live Cell ReadyProbes nuclear stain. The peeled sheet shows 3×3 regions of localized positive Gaussian curvature, bearing a striking resemblance to the cones formed by actuated liquid crystal elastomers. Seven of the nine cones are clearly visible in the figure, and the two missing cones are located near a corner that has folded or has a small tear due to liquid movement. More examples are shown in SI. The cross sections confirms that the curved cell sheets remain thin, even in the mounds, and that the mounds are not formed by accumulation of cells (Figure 3 and Figure S8.

Using the coefficients we obtained from fitting the flat sheet, we simulate a cell sheet featuring a 3×3 defect array. As in experiments, we observe mounds located at the center of topological defects, see Figure 3C and Movie S2. The XZ and YZ projections show slices through the central topological defect; both curvatures are peaked with the same sign indicating positive Gaussian curvature localized on the central defect. We can directly calculate the polyhedral Gaussian curvature of the simulated surface, which shows a clear 3×3 grid of localized positive Gaussian curvature aligned with the +1 defects, see Figure 3C (bottom right inset).

The generation of Gaussian curvature around a +1 topological defect can simply be understood by considering the change in radius and circumference of the material around the defect. Since the circumference is parallel to the alignment of the cells, we expect it to contract more than the radius. This change in the ratio between the circumference and radius of the patch of cells induces positive Gaussian curvature. For an infinitely thin material with constant λ and ν, the predicted final shape is a cone (schematic shown in Figure 3A(iii)). If we call ϕ the half‐angle of the cone, the steepness of the cone can be related to the contraction of the material by the equation $\sin\left(\phi \right)=\lambda^{1+\nu }$, see [40] for a derivation.

Instead of cones, however, both the cell sheets and simulations feature mounds with smoother curvature, as can be seen from the cross‐section in Figure 3B and Figure S8. This is not surprising and is due to three factors. First, the cell layer has a non‐negligible thickness, implying a finite bending energy which causes the cone tip to be smoother (as shown for example in [70]). Furthermore, the cell layer is soft, making it difficult to sustain the discontinuous shape of a cone. Moreover, the size of the defect core in cell monolayers is large compared to the small (ideally null) size of the topological defect core in liquid crystal elastomers. The defect core has zero S by definition, which implies anisotropic relaxation is reduced toward the center of a defect, thus λ and ν are not spatially constant 42.

We can estimate an effective value of λ and ν from the confocal microscopy images and compare them to those measured for the flat sheets. Figure 3D shows the box plots of λ and ν for cell sheets with uniform alignment, while the filled horizontal rectangles show the result of the simulations of a flat sheet. For a mound generated by a topological defect, we can estimate λ by taking the ratio between the circumference of the largest circular ridge around a topological defect in the initial pattern, and the circumference of a fully formed mound. The latter is calculated in experiment from the distance between two adjacent mound tops multiplied by π. This gives estimates of λ highly compatible with those that we measured for flat sheets. We then measure the half‐angle of the cone ϕ by directly measuring the steepness of the mounds, see Figure 3B. This can be combined with our estimate of λ to give an estimate of the Poisson ratio, which is again in good agreement with our measurements from flat sheets (purple circles Figure 3D). We take the same protocol with our simulated cell sheets and obtain estimates of λ=0.33 and ν=−0.72, again highly compatible with our estimates from experiments and simulated flat sheets. Note that ν is negative, as was also reported for other biological tissues such as tendons, arteries, and skin [71, 72, 73], and making artificial implants that mimic the Poisson ratio of biological material is a key challenge for tissue engineering to obtain realistic mechanical responses [74]. This auxetic material is unlike conventional liquid crystal elastomers, which typically expand perpendicular to the director.

The in‐plane contraction of the material dictates the presence of positive Gaussian curvature, but does not imply a particular sign of mean curvature. This means that a localized peak or valley are equally likely to form at the center of the +1 topological defect. In liquid crystal elastomers, the bending direction is typically determined by the position of the heating source or a tilted angle of molecular alignment on the surface [39]. In our experiments and simulations we observe bending in both directions within the same sheet, as shown in Figure S9. This implies that symmetry is not broken between the two sides of the cell sheet.

**FIGURE 4:**
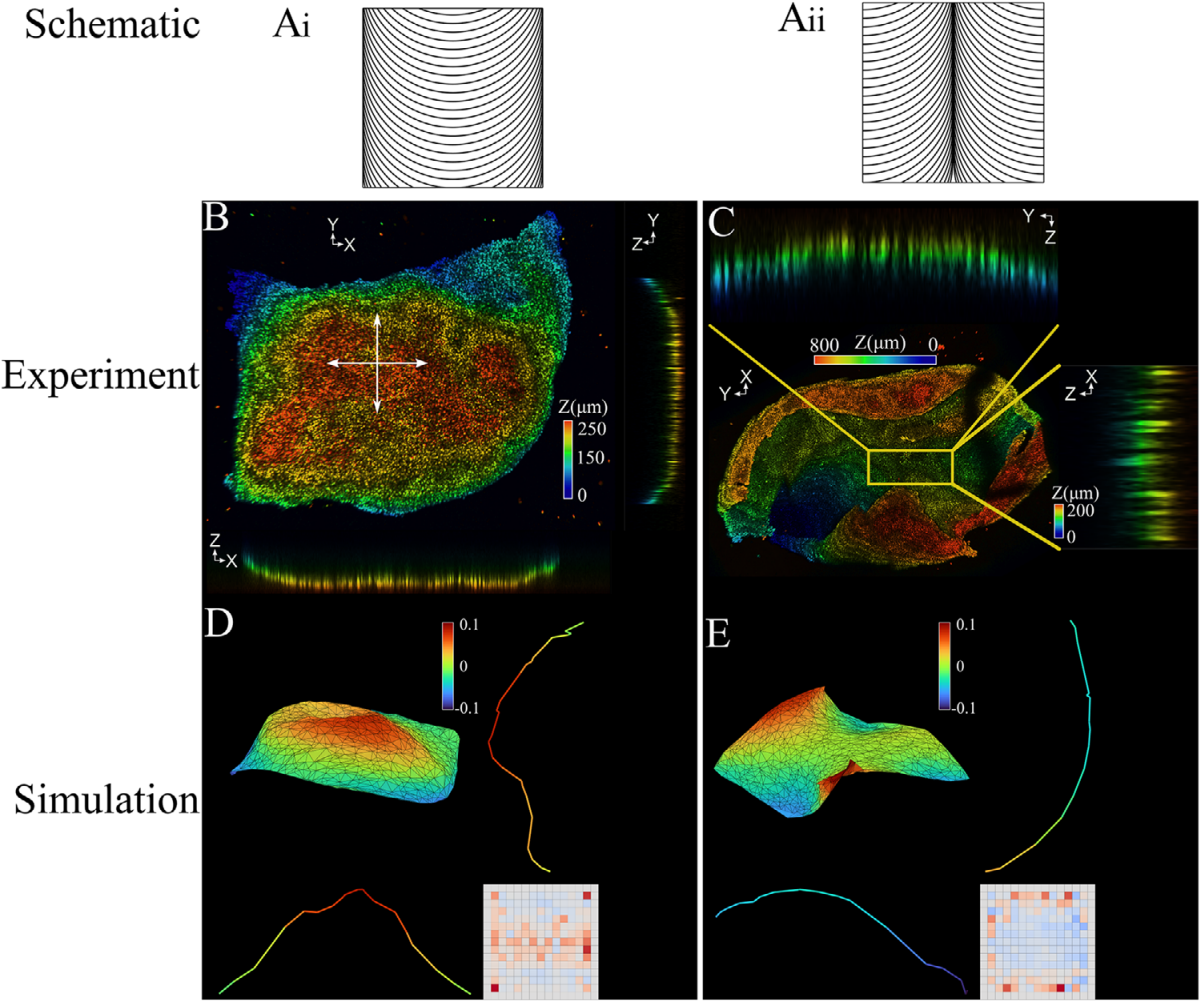
Actuation of cell sheets with non‐singular patterns. (A) Schematics of the tested alignment patterns featuring bend (left), and splay (right) patterns. All the patterns vary in 2‐D and are made of short ridges like the schematic in 3A (ii) with the height of the ridges at 1.5−2 μm, ridge width 9 μm. (B,C) Confocal microscopy image of a peeled sheet of cells from the bend (B), and splay (C) patterns. The overall size of the detached samples are 1800 μm× 3000 μm (B), and 1800 μm× 3000 μm (C). The color bar shows the height difference. The cross sections show the consistent thickness of the sheet, and the curvature along the two principal directions for the bend (B) and splay (C) patterns, respectively. (D, E) Same as previous row but for simulated cell sheets, akin to what was seen in 3C (see also Movies S3 and S4). The insets in the corner are the maps of the Gaussian curvature of the sheets averaged over multiple simulations, see Supporting Information for details.

While all experimental and simulation results shown so far are consistent with the behavior of anisotropic elastic materials, the higher density of cells at +1 topological defects [34, 64, 67, 75] may also play a role in generating the curvature [76]. Since the cells in the core of the +1 defects are packed to a higher density and already have a smaller size compared to cells in the surrounding sheet, it is possible that they cannot contract as much as the cells further away from the defect core. Furthermore, it has been shown that relaxing the liquid crystal energy around a +1 topological defect can also lead to cone shapes [77]. To rule out these alternative mechanisms and demonstrate that the cells' 3D structure is a consequence of their nematic order, we test two non‐singular patterns with constant cell density and order parameter that are able to generate Gaussian curvature in liquid crystal elastomers. These patterns are shown in Figure 4A(i,ii) and were previously studied by Mostajeran and coworkers [41]. We verify that we can indeed generate positive or negative Gaussian curvature in the center of the samples shown in Figure 4B,C respectively, and depicted from simulations in Figure 4D,E and Movies S3 and S4, as expected. In experiments, the edges of the samples tend to roll randomly, most likely due to the very soft and floppy nature of the elastic material. Nevertheless, this measurement shows that the actuation occurs and generates Gaussian curvature, even without the variation of cell density and order parameter associated with topological defects.

## 3 Discussion

There is much need for strategies to generate 3D cell cultures that mimic organs. Most of the existing strategies involve imposing curvature through geometric constraints [50, 78, 79]. This work demonstrates a different path to control curvature through cell alignment, using the spontaneous generation of Gaussian curvature that is characteristic of anisotropic elastic materials under frustration. Further, our method to detach cells do not require any thermal or chemical stimuli.

We demonstrate that during detachment, aligned sheets made of NIH‐3T3 fibroblasts contract anisotropically. Their contraction is greater along their nematic director than perpendicular to it, with a stretch factor λ≈0.3 and a 2D Poisson ratio ν≈−0.55. Because of this anisotropic contraction, we can generate 3D structures by spatially patterning the alignment of the cells in 2D. Concurrently with our work, Guillamat et al. [80] and Huang et al. [81] independently developed complementary experimental approaches, with results that are consistent with ours. Here we demonstrate anisotropic contraction in cell sheets patterned both with +1/–1 defect arrays (Figure 3) and with non‐singular patterns (Figure 4). All these results are supported by numerical simulations. The detached cell sheet is viable immediately after peeling and can be transferred to a new dish for regrowth.

The results raise the question of the mechanisms underlying cell contraction. While more research is needed in this area, previous work provides important context. For example, it was shown that the actomyosin‐dependent cortical remodeling in HeLa cells is mediated by proteins of the families ROCK and ERM, which promote loss of adhesion both during mitosis and upon rounding during interphase [51]. In fibroblasts, it is the disassembly of actin stress fibers that starts the process leading to cell shape change upon trypsination [82]. Other studies in mouse embryonic fibroblasts have shown that each cell can undergo a 40% reduction in cell volume in 10 min as the cells become round, and that this process is accompanied by a total loss of actin orientational order [83]. In addition, it was shown that actin remodeling in fibroblasts is key in maintaining connective tissue tension [52, 53].

All these studies suggest that cell contraction is in many cases an active process. Our results show, however, that the peeling process is robust, and that blocking the active remodeling of the cytoskeleton does not prevent the contraction of the monolayer upon peeling. We test the effect of sample exposure to (i) Y27632, which interferes with Rho‐Kinase pathway and prevents active remodeling of actin, (ii) mycalolide B, which favors actin depolymerization, (iii) cytochalasin D, which prevents the addition of actin monomers to existing filaments, (iv) a “freeze actin” mixture composed of a combination of latrunculin A, jasplakinolide and Y27632 (Figure S3). None of these treatments blocks peeling or prevents the cell sheets from contracting. The picture that emerges is that once the links between cells and substrates are broken, if the layer cohesion is not broken, the system relaxes to its final shape. The exact contribution of active contraction to the final shape needs further investigation.

Our qualitative explanation for the anisotropic contraction is as follows. Substrates with high stiffness have been shown to promote actin orientational order [65], and that the actin orientation is highly correlated with cell orientation. The substrates used in our experiments have a stiffness of ∼200 MPa, thus are characterized as stiff substrates. On plain substrates, where cells do not adopt a common orientation, actin filaments are distributed isotropically. This results in isotropic contraction upon detachment. In contrast, striped substrates impose a preferred orientation, leading to alignment of the actin filaments and extension of cells along this axis. Consequently, upon detachment the contraction perpendicular to the stripes is reduced resulting in an anisotropic response.

From the explanation above, however, one could expect that the contraction along the parallel direction is enhanced for uniformly aligned cells. This is not shown in our data. We believe that this depends on two factors. First, there is a residual alignment in the actin network after contraction, shown in the measurements of the order parameter, indicating that when the cells detach, they are unable to fully relax to an isotropic state. Moreover, cells grown on poly‐D‐lysine coating have a relatively low aspect ratio, which limits the detectable difference between uniformly aligned cells and randomly aligned cells. Indeed a higher parallel contraction was shown in mature fibroblasts grown on patterned fibronectin strips [21], which induced a much higher aspect ratio of the fibroblasts prior to detachment.

Our results indicate that, as for liquid crystal elastomers, it is the in‐plane alignment that determines the structure of the peeled cell sheet. There are however some important differences between the cell systems and liquid crystal elastomers. First, the detachment process is irreversible. While we have shown that cells survive the detachment and can be replanted, they do not stretch back to the initial shape but regrow from the edges of the contracted sheet. The system in this respect resembles more a one‐shot shape‐memory system. Moreover, in our dense cell sheets we never observe complete loss of actin orientational order, indicating a possible role of the matrix surrounding the cells or of cell‐cell interactions. The relaxation of the cell sheet is more complex than the free energy minimization in liquid crystal elastomers. However, the mechanics of the system can be understood using the same framework, as a change in mechanical properties related to a change in order parameter and the conceptual tools developed for LCE actuators are applicable here. Overall, more studies are needed, for example to clarify the role of cell aspect ratio and ECM development, but our work identifies a promising direction for fundamental studies of tissues as metamaterials that can achieve target shapes thanks to the nematic order of cells, and sets a step in linking nematic order to tissue morphogenesis.

Our findings make a strong case for the layer‐by‐layer approach to tissue engineering. The actuation of other cell types may look very different from the actuation of fibroblasts. First, the initial aspect ratio of the cells before peeling is important in determining whether the contraction is anisotropic or not, which in turn determines the ability to form 3D structures. Secondly, other cell types such as C2C12 myoblasts start forming multilayers after cell confluency [84], so that when they are ready to peel spontaneously they are not a monolayer anymore. However, even cells that contract isotropically and do not form 3D structures spontaneously may be induced to do so if they are supported by a fibroblast layer, highlighting the power of the layer‐by‐layer approach.

In conclusion, we have demonstrated a new tool to control Gaussian curvature in cell layers. This allows for the realization of complex 3D structures, needed for physiologically relevant experiments that mimic organs, starting from 2D cultures, which are easy to handle. This could potentially become a powerful tool in tissue engineering. In addition, this work is yet another piece of evidence of how nematic alignment and topological defects are mediators of morphogenesis.

## 4 Methods

### 4.1 Cell Culture

NIH 3T3 mouse fibroblast cells (ATCC CRL‐1658^TM^) are used in all experiments for peeling. C2C12 cells (ATCC CRL‐1772^TM^) were used for positive control for the collagen IV immunostaining. Both cell types were grown under identical conditions. The cells are cultured on Thermo Fisher Scientific Nunclon Delta surface‐coated dishes in 89% Dulbecco's Modified Eagle's Medium (DMEM) ‐ high glucose formulation (containing 4.5 g/L glucose, L‐glutamine, sodium pyruvate, and sodium bicarbonate; Sigma Aldrich), supplemented with 10% fetal bovine serum (FBS; Sigma Aldrich) and 1% penicillin‐streptomycin. The cells used in the experiments are between passages G9 and G15.

### 4.2 Substrate Manufacturing

Substrates are manufactured in a clean room using standard photolithography techniques on 10 cm silicon wafer chips. SU‐8 2 negative photoresist is used to reach the desired height of 1.5 to 2 μm for the designs and measured using a profilometer.

### 4.3 Substrate Preparation

Polydimethylsiloxane (PDMS; Sylgard 184, Dow Corning), mixed with 10% curing agent, is used as a substrate for cell experiments. An SU‐8‐coated silicon wafer is placed in a petri dish and coated with a thin layer (3–5 mm) of desiccated PDMS mixture (1:10 ratio). The petri dish is then desiccated again to remove any bubbles formed during pouring. After desiccation, the sample is cured on a hot plate at 90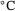 for 3–4 h. Once cured, the PDMS substrate is carefully removed.

This PDMS mold is then used to create negative molds with Norland Optical Adhesive 81 (NOA‐81), a UV‐curable glue. A flat‐bottomed glass petri dish containing 3–4 drops of NOA‐81 is prepared, and the PDMS mold was inverted onto it to ensure complete coverage of the patterned substrate with NOA‐81. The sample is degassed to ensure accurate replication of the pattern by the UV glue. The petri dish is exposed to UV light at 302 nm (8 W; Ultra Violet Products‐3UV) for 20 min. The petri dish is then flipped, and an additional 20 minutes of UV exposure is done to ensure complete polymerization of the UV glue. Finally, the dish is heated at 60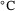 for 30 min. Once completed, the original PDMS mold is removed, leaving behind a negatively patterned substrate made of UV glue, which is then used to produce PDMS copies for experiments.

The patterned PDMS copies (parallel ridges, or integer defects) are cut into rectangular slabs with sides going from 2.5 to 7.5 mm and with one of the sides fixed around 7.5 mm, and cleaned using scotch tape. All these measurements have uncertainty of circa 1mm due to imprecision in cutting. For patterns with parallel ridges, the long side of the slab is parallel to the ridges. A thin layer (2 mm) of PDMS (1:10) is poured onto a plastic petri dish and cured overnight at 37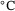. The petri dish along with the pattered PDMS substrate is treated with oxygen plasma (Harrick Plasma Cleaner) with RF power 30W for 3 min with a pressure of 300 mtorr. Once the plasma‐cleaned, the PDMS slab is attached to the plastic petri dish with the patterned side facing up and then heated for 1 min at 60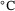.

The sample is then sterilized with ethanol and prepared for substrate treatment with Poly‐D‐Lysine hydrobromide (Sigma–Aldrich). The substrate is coated with 0.1 mg/mL concentration of Poly‐D‐Lysine in Milli‐Q Water, barely covering the surface. After 30 min the dish is cleaned with sterile Milli‐Q Water and allowed to dry for 90 min. Once dried the cells are planted on the substrate. The cell concentration of the suspension is determined using a hemocytometer with 10 μL demarcation. The concentration of cells used for plating the dish varies between 500–1000 cells/${\mathrm{mm}}^{2}$. We add a total of 7–8.5 mL of medium to ensure sufficient fluid height above the substrate.

### 4.4 Peeling Method

Cells are allowed to grow undisturbed for 2–4 days inside an incubator at 37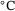 with 5% ${\mathrm{CO}}_{2}$. Once the cells are over‐confluent, the samples are carefully carried to the microscope so as not to induce spontaneous peeling. Once at the microscope, we use a scalpel with the sharp side of the blade touching the edge of the slab but pointing opposite to the scratching direction. Before using the scalpel, while the cells are still attached, the cell medium is reduced to less than 6mL in a 2‐inch petri dish. This helps prevent sloshing of fluid. When applying pressure to the edges of the PDMS, we made sure that we only applied a gentle pressure. With this, we scratch each edge of the PDMS slab. This is carried out for all edges, from one vertex to another ensuring the bonds of the cells from the edge of PDMS are carefully severed, while also not cutting into the PDMS. Once cut, the cells either spontaneously peel completely from the surface or get pinned at times as shown in Figure S2. In this case, a sharp‐end tweezer is used to cut the anchored cell, while observing through the microscope. To aid in peeling the same tweezers are also used to brush the cell sheet which is already peeling, while ensuring not to puncture the cell sheet. This results in a completely detached cell sheet from the substrate. The cells are slightly denser than water, so the cell sheet tends to settle on top of the substrate. For staining the cell sheet for actin, the cell sheets are pinned close to a corner of the cell sheet through to the substrate while peeling with a 30 μm needle, while being observed under the microscope. This helps prevent any movement of cell sheet while the staining protocol is carried out.

### 4.5 Fixing and Staining

To observe the cell nuclei, NucBlue Live Cell ReadyProbes (Hoescht 33342) stain is added to the dish at a concentration of 1 drop/ml of cell media and followed by 30 min of incubation.

Cells can also be fixed and observed either before or after peeling. For post‐peeling samples, as described in the previous section, a 30 μm needle is used to secure the unattached cell sheet to the substrate. All fixed samples undergo the same fixation protocol, using 4% Paraformaldehyde (PFA) in PBS Ready‐to‐Use Fixative (Biotium) to fix the cells sheet. Around 100 μL of PFA is applied over a 4 ${\mathrm{mm}}^{2}$ cell sheet and incubated for 20 min. The sample is then washed twice with 1x Phosphate‐buffered saline (PBS), leaving the PBS in the sample for 5 min during each wash. In some cases, a third wash was applied.

For actin staining, cells are permablilized by adding 50−100 μL of 0.1% (V/V) Triton X‐100 (Merck) in 1x PBS for 10 min, followed by two 5‐min washes with 1x PBS. To block non‐specific binding 50−100 μl of 1%(W/V) Bovine serum albumin (BSA, VWR) in 1x PBS is added and incubated for 1 hour, after which the samples are washed twice more with PBS 1x. Actin is tagged using 0.2% (V/V) Rhodamine Phallodin (invitrogen) diluted in 1x PBS. 50–100 μL of this solution is used to coat the sample and incubated for 1 hour before washing it again with 1x PBS. Following this, the cell sheet is stained using NucBlue Fixed Cell ReadyProbes, adding 2 drop/mL to 1x PBS in the petri dish, incubated for 20 min and washed again with 1x PBS.

### 4.6 Manipulation of Actin Cytoskeleton and Cells

To test the impact of actin cytoskeleton remodeling during the peeling process, we performed a number of peeling experiments where overconfluent cells before peeling were exposed to different drugs (i) inhibiting actin polymerization (30 min, 2 μM cytochalasin D [57]) (ii) depolymerizing actin (30 min 2 μM mycalolide B [56]), (iii) inhibiting active contraction (15–45 min, 10 μM Y27632 [55]), (iv) “freezing” actin [58] applying a combination of Y27632 (10 μM), the inhibitor of actin polymerization latrunculin B [85] (500 nM) and the inhibitor of actin depolymerization jasplakinolide [86, 87] (1 μM) for 30 minutes, (v) cleaving cell cell connections (40 s standard cell culture trypsin, Merck T4174) or (vii) crosslinking / fixing the biological sample (20 min in 4% paraformaldehyde in PBS). All treatments were performed under standard cell culture conditions (37°C, 5% CO2 in air, humidified atmosphere). To allow for multiple testing and control and test conditions for cells grown in the same dish, experiments where cells were pre‐peeling‐treated were performed on 7.5 mm × 5 mm PDMS with ridges, where each PDMS slab was cut into quarters, keeping the long edge parallel to the ridges.

### 4.7 Collagen IV Staining and Detection

Samples were grown as usual for peeling experiments on flat PDMS, and fixed 20 minutes at room temperature using 4% paraformaldehyde in PBS. Antigen retrieval was conducted in 10 mM sodium citrate buffer pH 6.0 with 0.2% Tween20, 20 min at 90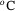. Unspecific binding was blocked for 1 hour in 1% BSA in PBS. The antibody against collagen IV was from Acris/Origene (cat ABIN113480, lot PN2051A, unfortunately discontinued, replaced by ABIN5596835 from Antibodies Online), used in a 1:400 dilution in PBS/0.2% BSA at 4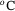 over night. Secondary antibody was a goat anti‐rabbit IgG conjugated to AlexaFluor568 (abcam ab175471) applied in a 1:500 dilution in PBS/0.2% BSA for 1 h at room temperature. The positive control was a C2C12 culture grown to 7 days post confluency on flat PDMS. Negative controls were “no primary controls” receiving only the secondary antibody. Stainings were visualized using the Nikon A1R confocal (see “Microscopy” section) using the 546 laser line.

### 4.8 Statistical Analysis

Statistical analyses are performed for the data shown in Figures 1 and 2. Comparisons in Figure 1D are calculated using a student T‐test with unequal variances. In Figure 2D, group comparisons are conducted using one‐way ANOVA, followed by pairwise Tukey‐Kramer test between samples from the same and different conditions. Statistical significance is set at p≤0.05, with p≤0.05, p<0.01, and p<0.001 denoted by ∗, ∗∗, and ∗∗∗, respectively. Sample sizes for each analysis are reported in the main text or Supplementary Information.

### 4.9 Microscopy

Nikon Tl‐Eclipse Widefield microscope and Kinetix Scientific CMOS camera (Teledyne Photometrics) are used to image cell sample, using phase contrast and fluorescent imaging in 2D. Large multi‐point image is used to capture the complete cell sheet and patterned substrate by translating the stage along a grid with 15% overlap between the frames.

Confocal imaging is done using the Nikon A1R confocal unit with Ti‐2 LFOV micorscope body, and with A1‐DUG hybrid 4‐channel multi detector. LU‐NV laser unit is used to excite the sample at 407nm and 514nm for nuclei and actin respectively.

### 4.10 Distance and Error Estimation

Images obtained from both the microscope are processed by using ImageJ Stitching plugin by passing them through an ImageJ Stitching plugin [60]. For Z‐Stack large image stitching, an ImageJ macro was written and used in conjuction with ImageJ Stitching Plugin to get final image. ImageJ was later used to the measure the various length of the cell sheet and the ridges.

To account for the irregular shape of the cell sheets before and after peeling, we take 5 measurements per side of the samples before and after peeling, evenly distributed across the sample. In some cases, samples have a partially rolled side, or an “ear” near a corner. These are clearly visible from the phase contrast images. In this case, we measure the local thickness of the rolled side and add it to the measured length.

The percentage contraction in x and y directions are calculated using

(1) $$\begin{array}{c} C_{X}=\frac{x_{i}-x_{f}}{x_{i}}\\ C_{Y}=\frac{y_{i}-y_{f}}{y_{i}} \end{array}$$

where $x_{i},y_{i},x_{f}\;\mathrm{and}\;y_{f}$ are the average initial (substrate or before peeling dimensions) and final (post peeling dimensions) of the cell sheet. Errors in estimation denoted by $\delta x_{i},\delta y_{i},\delta x_{f}\;\mathrm{and}\;\delta y_{f}$ is given by standard deviation of measured $x_{i},y_{i},x_{f}\;\mathrm{and}\;y_{f}$. With this the error in $C_{X}\;\mathrm{and}\;C_{Y}$ is given by

(2) $$\begin{array}{c} \delta C_{X}=\pm \left\{\frac{\delta x_{f}+\delta x_{i}}{x_{i}-x_{f}}+\frac{\delta x_{i}}{x_{i}}\right\}\frac{x_{i}-x_{f}}{x_{i}}\\ \delta C_{Y}=\pm \left\{\frac{\delta y_{f}+\delta y_{i}}{y_{i}-y_{f}}+\frac{\delta y_{i}}{y_{i}}\right\}\frac{y_{i}-y_{f}}{y_{i}} \end{array}$$

### 4.11 Order Parameter

In order to get a projection of a confocal image, ImageJ is used to get a Maximum intensity projection of actin images. The maximum intensity image is passed through OrientationJ (ImageJ plugin) with a local window size (σ = 5μm) to get the orientation of actin fibers, both before and after the peeling. The orientation is then used to calculate the local order parameter S [44]

(3) $$S=\sqrt{{\left\langle \cos2\theta \right\rangle }_{x,y\in W}^{2}+{\left\langle \sin2\theta \right\rangle }_{x,y\in W}^{2}}$$

where θ is the local nematic director and W the window. S is the largest eigenvalue of the 2‐dimensional nematic tensor. It varies from 0 to 1 and captures the degree of nematic alignment within the designated area. The local order parameter is taken over a 30μm window.

The global nematic order parameter S is computed from the spatial distribution of the local nematic director θ(x,y) by first constructing the global nematic order Q‐tensor

(4) $$\begin{array}{c} Q=\left(\begin{array}{cc} {\left\langle 2\cos^{2}\theta -1\right\rangle }_{x,y} & {\left\langle 2\cos\theta \sin\theta \right\rangle }_{x,y}\\ {\left\langle 2\cos\theta \sin\theta \right\rangle }_{x,y} & {\left\langle 2\sin^{2}\theta -1\right\rangle }_{x,y} \end{array}\right) \end{array}$$

where ${\left\langle \cdot \right\rangle }_{x,y}$ denotes the average over all positions in the field of view.

The eigenvector corresponding to the largest eigenvalue of Q defines the global nematic director, and the magnitude of this eigenvalue gives the global nematic order parameter S. The resulting values of S are shown in Figure S7.

### 4.12 Simulation Details

The simulation approximates the cell sheet with a point cloud, we approximate the internal stresses of the cell sheets as a set of springs connecting the points in the cloud. Note again, that the individual points and springs do not directly correspond to any part of the tissue. Over the course of the simulation, the rest lengths of the springs are adjusted to reflect the changing internal stresses. The points in the cloud are allowed to move to relax the stress accumulated in the springs, which approximates the changing shape of the cell sheet.

The motion of the points in the point cloud follow the overdamped Langevin equation:

(5) $${\underline{\dot{x}}}_{i}=\mu {\underline{F}}_{i}$$

where ${\underline{F}}_{i}$ is the force on point i.

The force on point i is calculated as the sum of all spring forces acting on i, which is written:

(6) $${\underline{F}}_{i}=k\sum_{j\in C_{i}}{\underline{\hat{r}}}_{ij}\left(l_{ij}-l_{ij}^{*}\right)$$

where ${\underline{\hat{r}}}_{ij}$ is the unit vector pointing from point i to point j, k is the spring constant, and $l_{ij}$ is the distance from point i to point j.

$C_{i}$ is the set of points connected to point i by a spring, which is determined by performing a 3D Delaunay triangulation on the point cloud. Finally, $l_{ij}^{*}$ is the rest length of the spring connecting point i and j. The rest length is calculated according to the following equation:

(7) $$l^{*}=l^{0}\sqrt{\alpha (1+\left(\beta -\gamma /2\right)\Delta S+\gamma \Delta S{\left({\hat{\underline{l}}}^{0}\cdot \hat{\underline{P}}\right)}^{2})}$$

Here $l^{0}$ represents the initial length of the spring, ${\underline{\hat{l}}}^{0}$ is the unit vector parallel to the initial spring. $\underline{\hat{P}}$ is a unit vector parallel to the nematic texture at the midpoint of the spring in its initial position, ΔS is the change in the order parameter at the midpoint of the spring.

The coefficients α, β and γ control how the rest length of the spring changes over the course of the simulation. α corresponds to a isotropic, uniform rescaling of the system, β corresponds to an isotropic rescaling of the system that depends on the local order parameter, and γ corresponds to an anisotropic rescaling of the system parallel to the nematic texture. Note here, γ is defined to be area preserving rescaling which is the reason that it occurs twice.

The stripes are described by the fields $\underline{\hat{P}}$ and ΔS, which we consider to depend on the x and y positions within the initial sheet only.

To initialize the simulation, n points are placed at random within a thin, flat sheet with dimensions $L_{x}$, $L_{y}$ and $L_{z}$. The point density is set to $n/\left(L_{x}L_{y}L_{z}\right)=10^{5}$. Prior to simulation, the positions of the points are then adjusted to reduce the coulomb energy associated with the point cloud. This ensures the points are roughly evenly spread out in three dimensions. The Delaunay triangulation is performed on this arrangement of points and $l^{*}$ is calculated for every spring according to Equation (7).

The forces on the points are calculated using Equation (6) which are used to iterate the positions of the points according to Equation (5). We rescale our time units by kμ and integrate Equation (5) using a Euler scheme with a timestep $\Delta t=5\times 10^{-2}$. Simulations were run for $10^{4}$ simulation steps in all cases. Maps of Gaussian curvature provided in Figures 3 and 4 were calculated by averaging over 10 simulations with different initial point clouds. For simulations used in Figure 1 we set $L_{x}=2L_{y}=100L_{z}=1$ and in Figure 3 and 4 we set $L_{x}=L_{y}=100L_{z}=1$.

### 4.13 Nematic Patterns for Simulation

The nematic patterns used in the simulations are as follows.

Figure 1F. $\underline{P}=\left[1,0\right]$

Figure 3, 4. We define a director angle θ such that $\underline{P}=\left[\cos\left(\theta \right),\sin\left(\theta \right)\right]$. We take ΔS=0.3 everywhere.

For the defect array (Figure 3), we divide the area into a 3×3 grid, the center of each square contains a defect. The defect array texture is the given by $\theta =\phi +\frac{\pi }{2}$ where ϕ is the polar angle around the center of the current grid square. The local change in order parameter is given by $\Delta S=0.3-\sum_{i}\exp\left(-r_{i}/\varepsilon \right)$ where the sum runs over the location of the center of each defect and $\varepsilon =L_{x}/100$ is the defect core radius.

For the bend texture (Figure 4 left) the texture is simply given by $\theta =-\pi /2+\frac{x\pi }{L_{x}}$. We take ΔS=0.3 everywhere.

For the splay texture (Figure 4 right) the texture is given by $\theta =\frac{x\pi }{L_{x}}$. We take ΔS=0.3 everywhere.

### 4.14 Simulation Parameter Fitting

We first take the uniform contraction in the absence of stripes to be $C_{∥}=0.66\pm 0.05$ and $C_{⊥}=0.69\pm 0.07$ from experimental data in Figure 1 in the manuscript. We simulate this configuration by setting ΔS=0, which leaves α as the only remaining free parameter. We can predict the value of α by fitting $C_{∥}$ with Equation 7 the following equation:

(8) $$C_{∥}=1-l/l_{0}=1-\sqrt{\alpha }$$

Where a value of α=0.1 corresponds to a prediction of $C_{∥}=0.68$.

In simulation with α=0.1 and ΔS=0, we obtain averaged over 10 simulations for different point clouds $C_{∥}=0.7$ and $C_{⊥}=0.7$ in good agreement with experiments, see Figure 1C.

By setting $\underline{\hat{P}}=\left[1,0\right]$ we introduce the uniform stripe pattern from Figure 1 in the manuscript; in experiments, this corresponds to a change of $C_{∥}\approx 0.7$ and $C_{⊥}\approx 0.5$. We fit each of these two data points individually. However, now we have three fitting parameters since ΔS≠0, this means that the solution is not necessarily unique.

(9) $$\begin{array}{cc} C_{∥} & =1-\sqrt{\alpha (1+\Delta S\beta +\Delta S\gamma /2)}\approx 0.7 \end{array}$$

(10) $$\begin{array}{cc} C_{⊥} & =1-\sqrt{\alpha (1+\Delta S\beta -\Delta S\gamma /2)}\approx 0.5 \end{array}$$

We retain the general rescaling parameter α=0.1, which, along with the first equation gives ΔSβ=−ΔSγ/2. This can be interpreted as fitting the 2D Poisson ratio of the change in shape. The second equation now allows us to fit ΔSγ=−1.6. Finally, we take ΔS=0.3 from Figure 2D which can be used to fix γ and β.

Simulating with these parameters gives good agreement with the experimental data, see Figure 1D. More precisely, we get averaged over 10 simulations for different point clouds $C_{∥}=0.7$ and $C_{⊥}=0.51$

### 4.15 Interpretation of Model Parameters

Following our experimental observations, we have identified three precursors that can influence the changing shape of the cell sheet; peeling, local change in alignment, and local orientation of the ridges. We can relate these to three distinct 2d deformation modes, which naturally arise from the symmetries of the precursors.

The simplest deformation is a uniform global rescaling, which would be described by a single scalar. We relate this to the peeling process, which is also global; in our experiments, the sheets is either peeled or not.

The next simplest deformation is local isotropic rescaling, which would be described by a scalar field. We relate this to the local change in order parameter, ΔS. As cells relax, they locally expand or contract in all directions.

Finally, we consider local anisotropic rescaling, which would be described by a vector field. This vector field indicates the orientation and degree of the local rescaling, which we relate to $\underline{\hat{P}}$ and ΔS, respectively.

The parameters α, β, and γ are simply the relative weighting of these three factors, and as such are dimensionless by design. They describe the local two dimensional shape change as a function of the three simplest deformation modes which share symmetry with the observables in our experiments. While higher order terms are allowed in the construction of the model they are typically of decreasing importance. When constructing a model in this way, one typically truncates the terms at a place that is justified by the observations. In this case, keeping the terms explicitly coupled to an experimentally observable field is well justified.

Our experimental observations are designed to isolate these three fields, allowing us to estimate them with high accuracy. Cells grown on a plain PDMS block do not have any induced order, thus there is no $\underline{\hat{P}}$ or ΔS field. In this case we see uniquely the effect of uniform, isotropic contraction, allowing us to fit α. Cells grown on uniform stripes have uniform $\underline{\hat{P}}$ and ΔS fields. This allows us to fit both β and γ from macroscopic measurements on the cells, namely $C_{⊥}$ and $C_{∥}$. Now that we know the relative importance of the deformation modes, we can test our model on more complex textures in which either one or both of $\underline{\hat{P}}$ and ΔS vary in space.

### 4.16 Characterization of Cones Formed from +1 Defects in Simulation

We measured the contraction λ and Poisson ratio ν for the defect array in Figure 3 in a similar manner as done for the experiments, here for the center cone of each simulation. λ is measured as the contraction of the whole surface which is the same as the contraction of the circle around the center cone. ν is calculated from estimating the mound as cone as in Ref. [40] with the half opening angle ϕ with $\sin\phi =\lambda^{1+\nu }$. We set a target radius at which we measure the height of the cone. This is done for different azimuthal angles around the cone. Only if the discretization point distance and the chosen target distance closely match, the ν is taken into account. The respective data is shown in Table S4.

## Conflicts of Interest

The authors declare no conflicts of interest.

## Supporting information


**Supporting Information**: adma73238‐sup‐0001‐SuppMat.pdf.

**Supplemental Movie 1**: adma73238‐sup‐0002‐MovieS1.mp4.

**Supplemental Movie 2**: adma73238‐sup‐0003‐MovieS2.mp4.

**Supplemental Movie 3**: adma73238‐sup‐0004‐MovieS3.mp4.

**Supplemental Movie 4**: adma73238‐sup‐0005‐MovieS4.mp4.

## Data Availability

The data that support the findings of this study are available from the corresponding author upon reasonable request.
