# Supplementary material for: Actuation of Cell Layers in Three Dimensions
Source: Adv Mater. 2026 May 13;38(34):e22191. doi: 10.1002/adma.202522191 (PMC13274700; doi:10.1002/adma.202522191)
Supplement: Supplementary file 1 — Supporting Information: adma73238‐sup‐0001‐SuppMat.pdf. [file ADMA-38-e22191-s005.pdf]

Actuation of cell layers in three dimensions

Kirsten Endresen[1*†], Aniruddh Murali[2†], Birte C. Geerds[3], Grecia M. Valenzuela Portillo[2], Maria Bloksgaard[2], Daniel J.G. Pearce[3*], Francesca Serra[2*]

[1]Dept. Physics and Astronomy, Johns Hopkins University, Baltimore, 21218, MD , USA.
[2]Dept. Physics, Chemistry and Pharmacy, University of Southern Denmark, , Odense, 5230, Denmark.
[3]Dept. Theoretical Physics, University of Geneva, Geneva, 1205, Switzerland.

*Corresponding author(s). E-mail(s): kirsten.d.endresen@gmail.com; daniel.pearce@unige.ch; serra@sdu.dk;
Contributing authors: anim@sdu.dk; birte.geerds@unige.ch; grecia@sdu.dk; mbloksgaard@sdu.dk;
†These authors contributed equally to this work.

1 Supplementary Information

Supplementary Figures

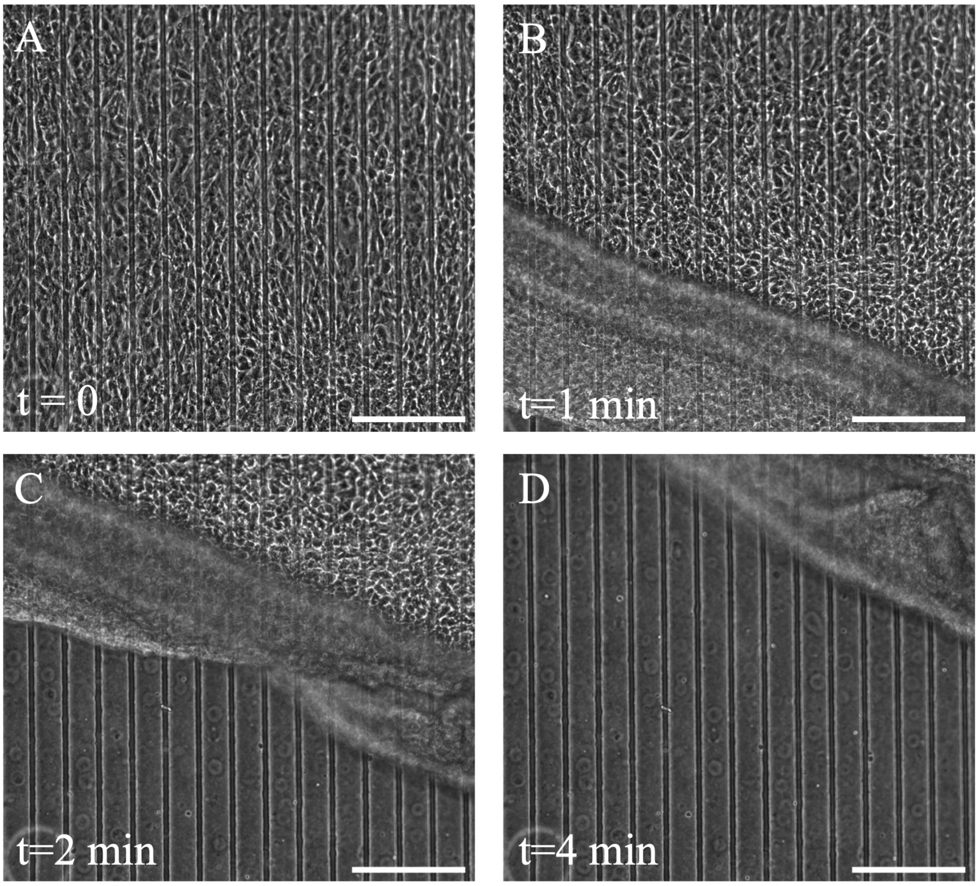

Figure S1 Progression of peeling cell sheet over 4-minute period. (A) At *t=0*, or just before scraping the edges of the cell layer, cells are attached to the PDMS substrate, which is patterned with h=2μm stripe ridges with w=60μm spacing. The cells are aligned and elongated along the direction of the stripes. (B) At *t=1* min after scraping the edges, the cells are starting to peel from the substrate. The peeling front has reached the middle of the image. The fully lifted part of the cell sheet is visible near the bottom of the image (C) At *t=2* min, the peeling front has traveled to the top of the image. (D) After *t=4* min, the cells within the frame are almost fully peeled. Scale bars are 200μm.

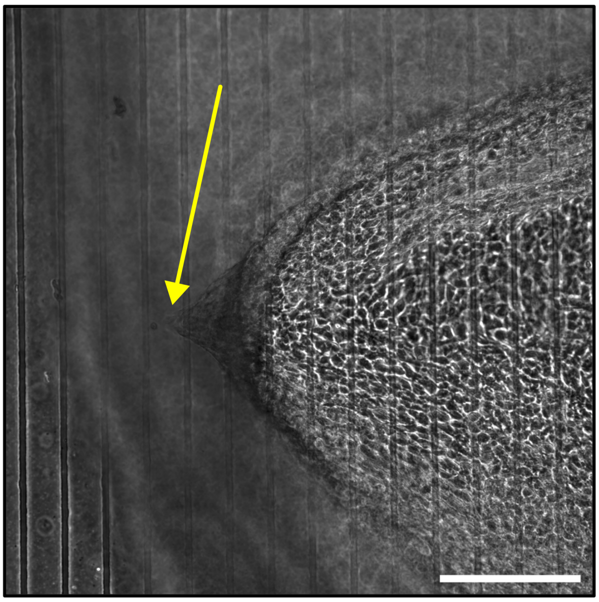

Figure S2 Pinning points, locations where the cell sheet is strongly pinned to the PDMS, occur during the peeling process. Here, the peeling front is traveling from the left of the image. The cell sheet remains attached to the substrate at this pinning point, as the peeling front deforms around it. The pinning point is observed through the lifted part of the cell sheet and is indicated by the arrow. The part of the cell sheet to the right of the pinning point has not yet peeled. Scale bar is 200μm.

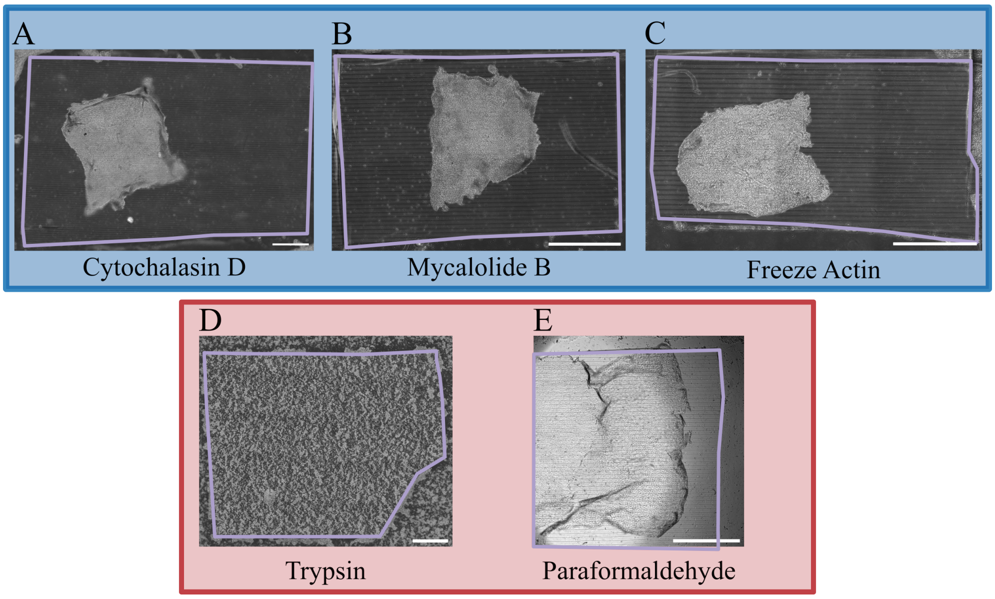

Figure S3 Effect of drugs on the cells' peeling. Phase-contrast image of peeled cell sheet after exposure to drugs. NIH-3T3 cells were grown on a PDMS substrate (highlighted with a purple outline) with stripes coated with poly-D-lysine. The blue color indicates treatment with drugs that modulate actin polymerization, which still allow for cell contraction and cell sheet cohesion. The red color indicates drugs that compromise cell cohesion (trypsin) or contraction (paraformaldehyde). Before peeling, cells were exposed to the following drugs: (A) cytochalasin D (30 min, 2 μM), which inhibits the attachment of monomers to actin filaments; (B) mycalolide B (30 min 2μM), which depolymerizes actin; (C) a "freeze actin" cocktail (JLY cocktail) with Y27632 (30 min, 10 μM), which inhibits actin remodeling, latrunculin (30 min, 500 nM) which prevents monomer attachment and jasplakinolide (30 min, 1μM), which blocks depolymerization of actin; (D) trypsin (40 s, standard cell culture), which cleaves all peptides (one can see each cell rounding up and the cell sheet loosing cohesion); (E) paraformaldehyde, which fixes the cells and makes the layer glassy and brittle. All scale bars are 1 mm.

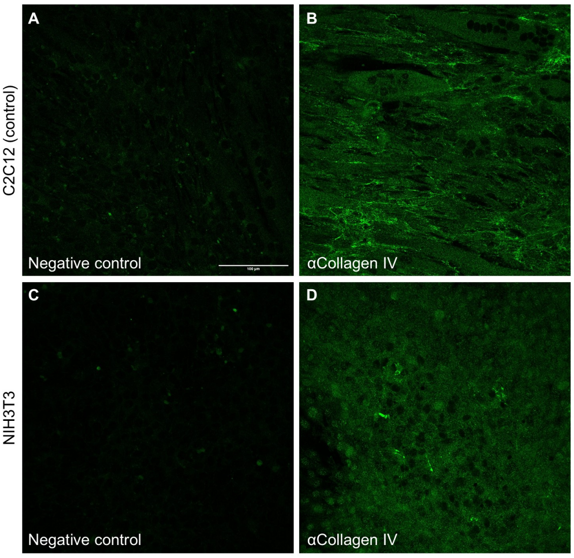

Figure S4 Collagen IV in C2C12 and NIH-3T3. The presence of collagen IV is visualized using immunocytochemistry. C2C12 cultures grown to 7 days post confluency are used as positive control. (A) C2C12 negative control (no primary antibody); (B) C2C12 showing clear collagen IV matrix between the cells; (C) NIH-3T3 negative control (no primary antibody); (D) NIH-3T3 stained for collagen IV, showing sparse to absent collagen IV matrix around the cells. Scale bar (100 μm) is the same in all panels.

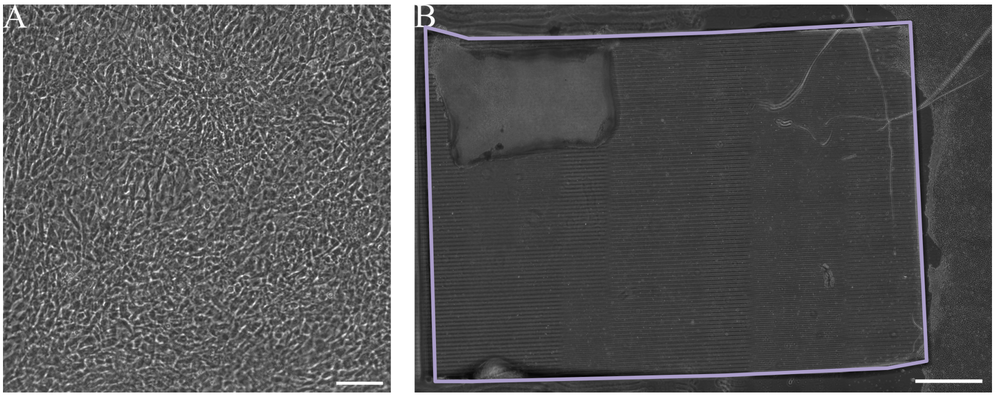

Figure S5 Example of peeling on plain PDMS substrate (A) Phase-contrast image of cells grown on a plain PDMS substrate coated with poly-D-lysine, prior to peeling. Scale bar, 100 μm. (B) NIH-3T3 seeded on a plain PDMS substrate (outline in purple) and peeled after 3 days of growth. Scale bar, 1000 μm. The stripes that are visible in the figures are actually located on the bottom of the substrate and not on the surface on which the cells are grown.

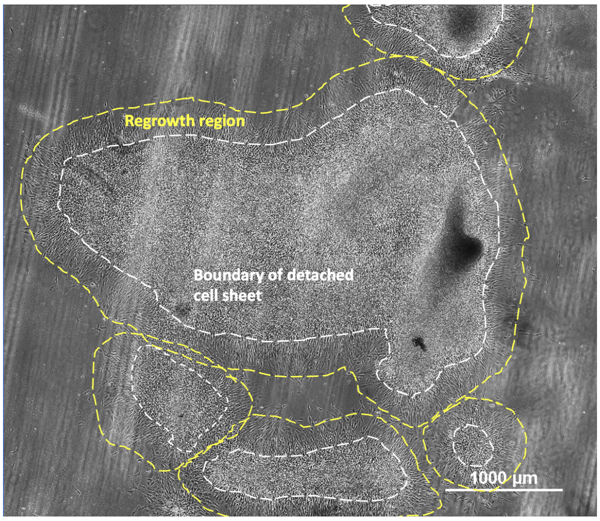

Figure S6 Regrowth of cells after detachment. A previously detached cell sheet reattaches and grows in a plastic Petri dish coated with fibronectin. Cells in the regrowth region are oriented radially outward from the cell sheet. The image is obtained after 1 day of regrowth inside a cell incubator microscope stage (Okolab Stage Top Incubator H-301K). The boundary of the detached cell sheets are identified as regions with high density of compact cells. The figure demonstrates that the cells are viable after peeling.

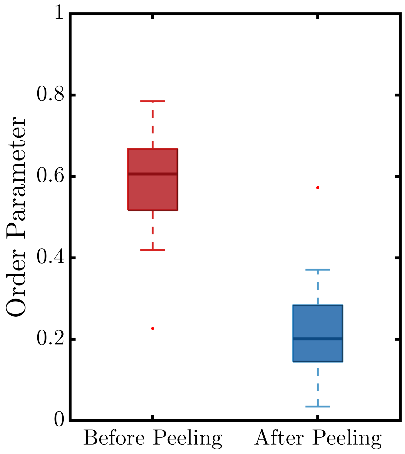

Figure S7 Global order parameter. Box plot of global order parameter as defined in the methods, and calculated over 2 different samples (total n=21 images) before peeling and 2 different samples (total n=31 images) after detachment.

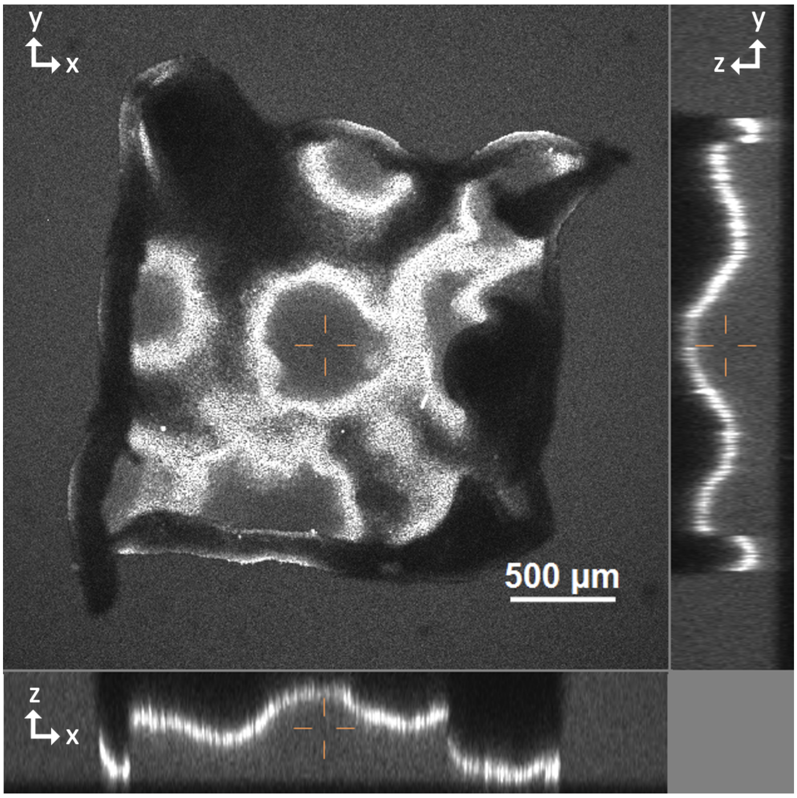

Figure S8 Confocal microscopy image of a cell sheet after peeling off from a defect array pattern, shown in Figure. 3A.i of the main paper. On the sides, the orthogonal view is shown, measured along the lines indicated by the cross in the figure. From the orthogonal view it is possible to see that the cell sheet, evident as the bright white profile, remains a thin layer after peeling.

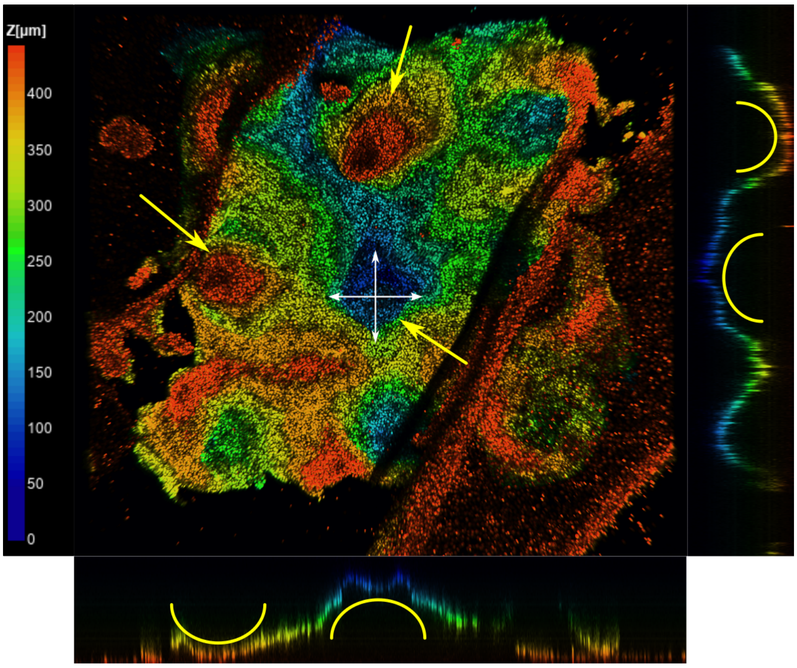

Figure S9 Confocal microscopy image of a cell sheet after peeling off from a defect array pattern, shown in Figure. 3A.i of the main paper. The 3D projection of the image illustrates the cusp and bowl at the location of +1 topological defect, indicated by the yellow arrows. On the sides, the orthogonal view is shown, measured along the lines indicated by the cross in the figure. The two projections clearly show a cusp and a bowl (indicated by yellow half circles), suggesting it is random with the out-of-plane deformation of the cell sheet.

Supplementary Table

Table S1 Parameters for the data points (orange squares) in Figure. 1.D for cell layers peeled from plain PDMS slabs. We report: the seeding density of the cells in cells/mm^2, the days of growth before peeling, the aspect ratio of the PDMS slab, and the contractions parallel and perpendicular to the x direction.

Sample	Seeding Density	Days of Growth	Aspect Ratio	$C_{\parallel}$	$C_{\perp}$
Sample 1	500	3	1.2	0.60 ± 0.08	0.60 ± 0.05
Sample 2	1000	3	1.5	0.62 ± 0.02	0.66 ± 0.03
Sample 3	500	4	1.5	0.66 ± 0.04	0.70 ± 0.05
Sample 4	1000	3	1.5	0.70 ± 0.03	0.75 ± 0.03
Sample 5	1000	3	1.5	0.71 ± 0.04	0.77 ± 0.04

Table S2 Parameters for the data points (purple triangles) in Figure. 1.D for cell layers peeled from striped PDMS slabs. We report: the seeding density of the cells in cells/mm^2, the days of growth before peeling, the aspect ratio of the PDMS slab, and the contractions parallel and perpendicular to the nematic director.

Sample	Seeding Density	Days of Growth	Aspect Ratio	$C_{\parallel}$	$C_{\perp}$
Sample 1	1000	3	1.5	0.76 ± 0.02	0.57 ± 0.05
Sample 2	1000	2	1.5	0.77 ± 0.01	0.52 ± 0.05
Sample 3	500	3	1.5	0.74 ± 0.02	0.53 ± 0.07
Sample 4	500	4	1.5	0.74 ± 0.01	0.56 ± 0.05
Sample 5	500	3	2	0.64 ± 0.03	0.58 ± 0.09
Sample 6	500	3	1	0.70 ± 0.04	0.60 ± 0.04
Sample 7	500	3	1.6	0.68 ± 0.03	0.59 ± 0.04
Sample 8	500	3	1.5	0.65 ± 0.03	0.61 ± 0.03
Sample 9	500	3	2.8	0.70 ± 0.02	0.48 ± 0.07
Sample 10	500	3	2.5	0.73 ± 0.03	0.38 ± 0.08
Sample 11	500	3	2.5	0.75 ± 0.03	0.49 ± 0.07
Sample 12	500	3	2.9	0.68 ± 0.02	0.36 ± 0.07
Sample 13	500	3	2.8	0.69 ± 0.01	0.46 ± 0.10
Sample 14	500	4	2	0.67 ± 0.02	0.44 ± 0.05
Sample 15	500	4	1.9	0.66 ± 0.03	0.41 ± 0.03
Sample 16	500	4	1.4	0.66 ± 0.02	0.48 ± 0.04
Sample 17	500	4	1.6	0.66 ± 0.02	0.48 ± 0.04
Sample 18	500	4	1.5	0.66 ± 0.03	0.45 ± 0.05
Sample 19	500	4	1.6	0.66 ± 0.02	0.48 ± 0.04

Statistical Analysis

We perform statistical analyses to assess the differences in the data shown in Figure 1 and Figure 2. A student T-test with unequal variance is used to compare the different conditions of contraction shown in SI Table.S1, and SI Table.S2. Statistical significance is defined at $p \leq 0.05$, with $p \leq 0.05$, $p < 0.01$, and $p < 0.001$ denoted by $*$, $**$, and

$***$, respectively. The corresponding p-values obtained via the T-test for the different comparisons are listed below.

Table S3 T-tests performed for different group comparisons.

Variable 1	Variable 2	p-value	Significance
**Plain and Striped (5 and 19 samples)**			
$C_{\parallel}$ Striped	$C_{\parallel}$ Plain	0.16	*ns*
$C_{\perp}$ Striped	$C_{\perp}$ Plain	< 0.001	$***$
**Striped $C_{\parallel}$ and $C_{\perp}$(19 samples)**			
$C_{\parallel}$ Striped	$C_{\perp}$ Striped	$<< 0.001$	$***$
**Aspect Ratio > 2 and < 2 (5 and 11 samples)**			
$C_{\parallel}$ $AR > 2$	$C_{\parallel}$ $AR < 2$	0.54	*ns*
$C_{\perp}$ $AR > 2$	$C_{\perp}$ $AR < 2$	0.016	$*$
**3-Day and 4-Day growth, striped substrate (11 and 7 samples)**			
$C_{\parallel}$ 3 Day	$C_{\parallel}$ 4 Day	0.097	ns
$C_{\perp}$ 3 Day	$C_{\perp}$ 4 Day	0.203	ns

For the local order parameter shown in Fig.2.D, we combine values from two samples, each comprising multiple images taken across the entire substrate, and detect a significant difference between the local order before and after peeling ($p < 0.001$). To illustrate reproducibility, we also plot the local order parameter for each sample individually, both before and after peeling, in Supplementary Fig. S10. Group comparisons are performed using one-way ANOVA followed by post hoc analysis with the Tukey–Kramer test. We can see there is a clear difference in local order measured before and after the peeling process in the induced between the samples.

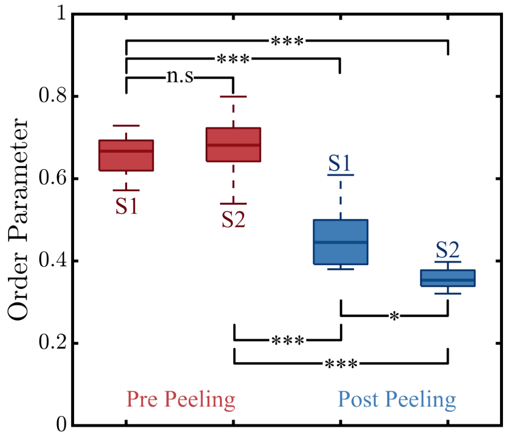

Figure S10 Sample-wise Order Parameter. Box plot of local order parameter as defined in the methods, calculated for each of the two samples used before and after peeling, and compared using one-way ANOVA, and post hoc analyzed with Tukey-Kramer test. Statistical significance is set at $p \leq 0.05$, with $p \leq 0.05$, $p < 0.01$, and $p < 0.001$ denoted by $*$, $**$, and $***$, respectively

Characterization of cones formed from +1 defects in simulation

For different simulations, values for ν and λ can be found in Table S4. On average we found $\lambda = 0.328$, $\nu = -0.718$.

Table S4 Contraction λ and Poisson ratio ν for different simulations.

λ	ν
0.328	-0.699
0.329	-0.69
0.346	-0.933
0.316	-0.646
0.322	-0.622

Supplementary Movie Captions

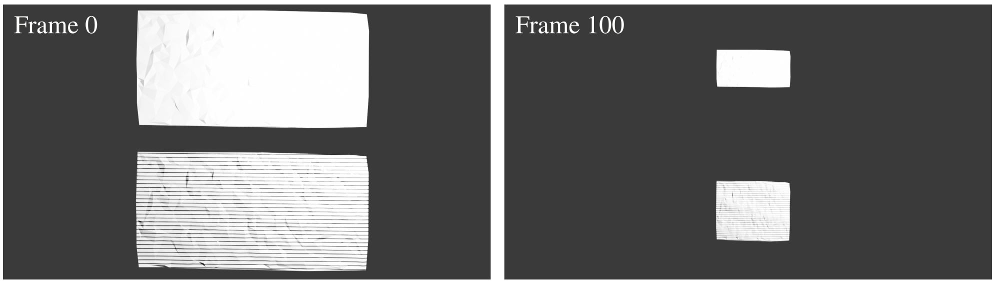

Figure S11 Movie S1. This movie shows the simulated difference between systems with and without uniform stripe patterns. The lines on the bottom surface indicate the embedded orientation from the textured substrate. The black lines fade over time indicating the loss of orientation order as the surface contracts. This simulation represents cells grown on a uniform ridged substrate, as in Fig. 1 (Main Text). It is simulated with parameters $\alpha = 0.1$, $\Delta S\beta = 0.8$ and $\Delta S\gamma = -1.6$. The top surface is simulated with no stripes, representing surfaces grown on plain substrates. It is simulated with parameters $\alpha = 0.1$, $\Delta S\beta = 0$ and $\Delta S\gamma = 0$. In both simulations, the surfaces contract and remain flat. The surface with lines contracts less in the perpendicular direction, which is apparent from the changing aspect ratio of the surface. The plain surface is simply rescaled without a change in shape.

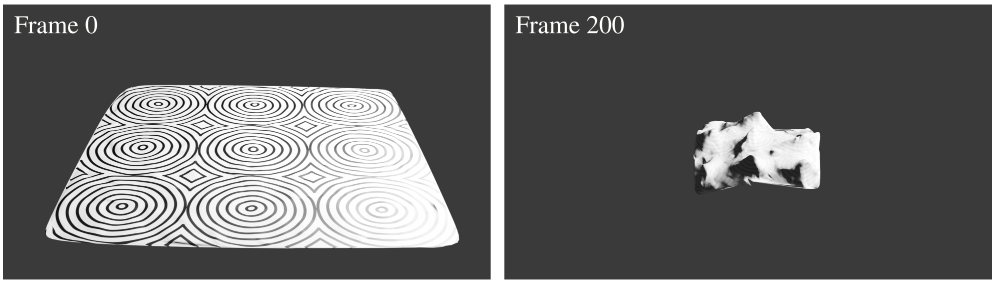

Figure S12 Movie S2. This movie shows a simulated cell sheet on the defect array texture shown in Fig. 3 (Main Text). The black lines indicate the embedded orientation from the textured substrate. The black lines fade over time indicating the loss of orientation order as the surface contracts. The emergence of peaks or troughs centered on the topological defects is clearly visible. This simulation is performed with parameters $\alpha = 0.1$, $\Delta S\beta = 0.8$ and $\Delta S\gamma = -1.6$ far from the defects. The orientation of the stripes, and the variation of ΔS close to the defect cores is given in the methods section of the manuscript.

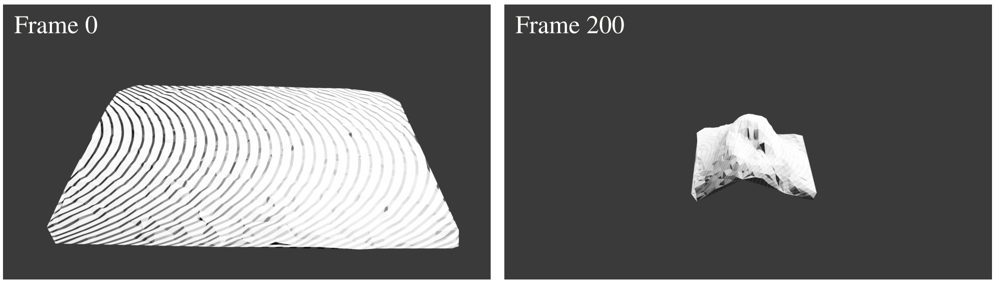

Figure S13 Movie S3. This movie shows a simulated cell sheet on the bend dominated nematic texture shown in Fig. 4A (Main Text). The black lines indicate the embedded orientation from the textured substrate. The black lines fade over time indicating the loss of orientation order as the surface contracts. The emergence of global positive Gaussian curvature is immediately apparent. This simulation is performed with uniform parameters $\alpha = 0.1$, $\Delta S\beta = 0.8$ and $\Delta S\gamma = -1.6$. The orientation of the stripes is given in the methods section of the manuscript.

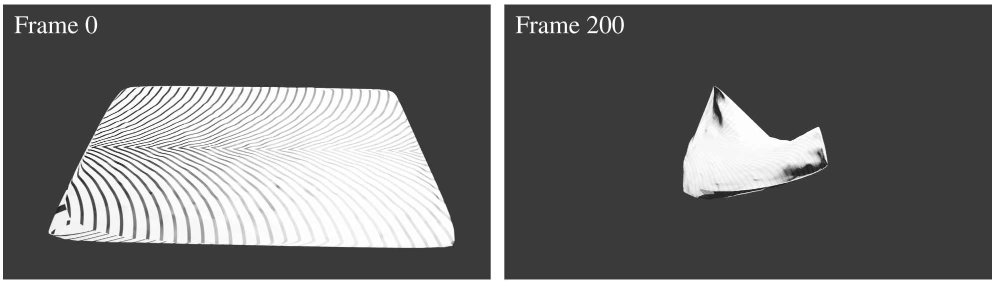

Figure S14 Movie S4. This movie shows a simulated cell sheet on the splay dominated nematic texture shown in Fig. 4B (Main Text). The black lines indicate the embedded orientation from the textured substrate. The black lines fade over time indicating the loss of orientation order as the surface contracts. The emergence of global negative Gaussian curvature is immediately apparent. This simulation is performed with uniform parameters $\alpha = 0.1$, $\Delta S\beta = 0.8$ and $\Delta S\gamma = -1.6$. The orientation of the stripes is given in the methods section of the manuscript.
